# Postnatal Care Utilization and Associated Factors among Mothers who gave Birth in the Aysaeta District, Northeast Ethiopia: A Community Based Cross-sectional Study

**DOI:** 10.4314/ejhs.v32i6.9

**Published:** 2022-11

**Authors:** Mohammed Ahmed Ibrahim, Kusse Urmale Mare, Mohammed Nur

**Affiliations:** 1 School of Public Health, College of Medical and Health Sciences, Samara University, Samara, Ethiopia; 2 School of Nursing, College of Medical and Health Sciences, Samara University, Samara, Ethiopia; 3 Afar Region Health Bureau, Regulator Team Coordinator, Samara, Ethiopia

**Keywords:** Postnatal care, Postnatal care service Utilization, Afar Region, Ethiopia

## Abstract

**Background:**

Postnatal care is given to mothers and newborn babies within 42 days of delivery. It is a period of high maternal and newborn mortality and is also the most neglected in terms of maternal health services in many parts of the world. This study aimed to assess postnatal care and associated factors among mothers who gave birth in the year preceding the survey of the Ayssaeta district.

**Methods:**

A community-based cross-sectional study was conducted among 406 mothers who gave birth in the year preceding the survey from August 02–30, 2020. Bivariable and multivariable logistic regression analyses were done to identify factors associated with postnatal care utilization.

**Results:**

Slightly greater than four out of ten mothers have visited postnatal care units at least once. Living in urban areas, giving birth in a health facility, having complications during labor and after, and getting advice during antenatal care visits were associated with higher odds of postnatal care utilization.

**Conclusion:**

Less than half of the mothers received postnatal care following the delivery of their last child. Living in an urban, place of delivery, experiencing labor and postpartum complications, and receiving postnatal care advice during antenatal care have affected the utilization of postnatal care. Promoting skilled delivery and antenatal care with a focus on rural areas can help mothers learn about postnatal care and increase the number of mothers who use it

## Introduction

Postnatal care is provided to mothers and newborn babies within 42 days of delivery. The postnatal period is a critical stage in the lives of both mothers and newborn babies. During this time, the majority of mother and newborn deaths occur (1). It is widely acknowledged as the most critical component of maternal and newborn health care in terms of reducing physical and cognitive deficits, as well as disability and death resulting from postnatal causes(1, 2). However, this is the least underutilized period for providing high-quality services (1).

Maternal mortality is a significant concern globally. There were 295,000 maternal deaths around the world in 2017. 99% of these deaths occurred in developing regions. Every day, approximately 810 women die as a result of complications related to pregnancy and childbirth. The major causes are hemorrhage, high blood pressure, and sepsis (3, 4).

The majority of maternal and infant deaths occur during the first month of birth, and over half of postnatal maternal deaths occur during the first twenty-four hours of delivery(5). They are also postpartum complications that are more likely to happen when delivered at home unless safe family practices are followed and health professionals are available(6, 7), and even though newborns die, the global reduction in infant mortality is slower than the reduction in under-five mortality (3).

Early postnatal care visits are one of the most crucial interventions in maternal health care for reducing morbidity and mortality. According to studies, only 48% of mothers follow up within two days of childbirth (8). and 36% of mothers in low-income countries receive at least one PNC within 42 days,(9). Only one-third of Sub-Saharan African women give birth in a facility, and less than one-fifth of women who give birth at home receive PNC visits within two days (10).

Studies show that PNC service utilization is influenced by a lack of awareness or no perceived need for postnatal care by women and their families; distance to the health facility (11, 12); meager transportation infrastructure, poor quality of service demand, and supplies in health facilities, prevailing traditional or cultural practices; and low decision-making power of women, the lifestyle of the community are among the common contributing factors (13, 14). Other reasons include lack of access to skilled birth attendants, and life-saving basic and comprehensive emergency obstetric and newborn care services are often limited or unavailable (7, 11, 12, 15).

Ethiopia's maternal mortality rate has decreased over the past two decades, but it is still very high at 412 maternal deaths per 100,000 live births, and the majority of these deaths occur after childbirth (12). According to the Ethiopia Demographic Health Survey (EDHS) report in 2019, PNC service utilization is extremely low in Ethiopia, with only 33.8% of women receiving PNC during the first two days after birth and 23.1% of Afar regional statistics (11).

The Federal Ministry of Health designed a health sector plan, the Health Sector Transformational Plan (HSTP) is aimed at improving equity, coverage, and utilization of essential health services; improving the quality of health care, and enhancing the capacity of the health sector at all levels of the system (16). The government's target is to reduce the maternal mortality ratio to 70 % by 2030 (17). Various strategies are used to reduce maternal mortality. Comprehensive postnatal health packages for mothers and babies are part of the health extension program, which is one of the approaches (18).

Postnatal care is the most useful maternal health service to improve the survival of mothers and their babies, but it is also the most neglected service. Although earlier research has focused on antenatal and delivery service utilization, less is known about postnatal care. There is a paucity of national and Afar region research on postnatal care. this study's aimed to assess postnatal care utilization and associated factors among mothers who gave birth in the one year preceding the survey in one year preceding the survey the Ayssaeta district of the Afar Region States, Northeast Ethiopia in 2020.

## Methods

Study setting, design, and period: A cross-sectional study was conducted in the Aysaeta district in the Afar Region State from August 02–30, 2020. The total population of the district is estimated to be 55,293 and the district has been subdivided into 2 urban and 11 rural kebeles (19). It has one functional hospital, two health centers, five health posts, and five private clinics.

**Sample size determination**: A sample size of 414 was determined by using a single population proportion formula and considering postnatal care service utilization of 57.5% (P) taken from a study done previously (20), 95% confidence level, margin error of 5% (d), and a 10% non-response rate.

**Sampling procedure**: A total of 4 randomly selected kebeles (1 from urban and 3 from rural) were included in the study. Using the health post registry, mothers who had given birth in the year before the survey were found at health posts. After identifying the number of eligible mothers in each kebele, the total sample size was proportionally allocated to the number of eligible mothers. A simple random sampling method was employed to select study subjects in each of the selected kebeles. Neighborhoods with an eligible mother were chosen by a lottery to be included in the study for households with more than one eligible mother. Revisits were made three times in cases where eligible respondents were not present at the time of the survey or if they were considered non-respondents..

**Study variables and definitions**: Postnatal care utilization was the outcome variable in this study. The variable was turned into a “Yes” or “No” answer based on whether or not the mother went to a healthcare facility within six weeks of giving birth. Socio-demographic variables:- mother's age, residence, marital status, religion, maternal and husband's education and occupation, family size, having radio and/or television, and deciding their own healthcare. The obstetric variables included age at first marriage, gravidity, parity, last planned pregnancy, ANC during last pregnancy, number of ANC visits, gestational age at first ANC, discussion of PNC during ANC, place of delivery, mode of delivery, ANC during last pregnancy, history of postpartum complications, and advice during PNC follow-up

**Data collection tool**: structured interviewer-administered questionnaire was developed and modified from the Ethiopian Demographic Health Survey (12) and relevant literature. and data were collected via face-to-face interviews.

The questionnaires contained The questionnaires contained socio-demographic characteristics obstetric information, and history of maternal health care consumption.

The data gathering instrument was initially developed in English, then translated into Afar-afar and Amharic (local languages), and finally back into English. As data collectors and supervisors, one health officer with a bachelor's degree and eight female registered nurses were recruited. The principal investigator researcher taught the data collectors and supervisors about the study's goal, sampling, and how to collect data over the course of three days.

**Data quality control**: To assure the quality of the data, the following measures were undertaken. Attempts were made to make the data collection tool tsuit the local language. Amharic version questionnaire was made available for the respondents having difficulty understanding Afar-Af language. Two weeks prior to the actual data collection, questionnaire was pretested on 5% of the sample size in the kebeles other than the sampled kebeles to assure the clarity and understandability of the instrument. Data collectors and supervisors were trained for three days on the data collection instrument and data collection procedures. During the actual data collection process, supervisors checked the questionnaires for consistency and completeness on a daily basis. Data collectors were female healthcare professionals with BSc degrees from out-of-study districts. After data collection, each questionnaire was given a unique code by the principal investigator.5% of the entered data were re-checked by comparing the entered data with the actual questionnaire. Moreover, frequencies were used to check for missed values and outliers, and corrections were made after revision of the original data using the questionnaire code.

**Data processing and analysis**: The SPSS version 20.0 statistical software was used to analyze the data, and the results were summarized and presented in text and tables. Binary and multivariable logistic regressions were used to analyze postnatal care utilization and associated factors with a P-value less than 0.025 were selected for further analysis by multivariable logistic regression. The odds ratio with a 95% confidence interval was used to determine the possible associations and their statistical significance.

**Ethical considerations**: A letter of ethical approval was obtained from Samara University's Research Ethics Committee. REF: EC0068/December 12th, 2019. A formal letter of permission to conduct fieldwork was also obtained from Afar Regional Health Bureau and administrative office. Each respondent gave written informed consent before being interviewed, and confidentiality was kept at every level of response in this study.

## Results

**Socio-demographic characteristics**: A total of 406 eligible women were successfully interviewed, yielding a response rate of 98.1%. More than half of the respondents were rural residents 209 (51.5%). The majority, 385 (94.8 %), were married, and 376 (92.6%) were Muslim by religion. The minimum and maximum ages were 23 and 34 years, respectively. The majority of mothers who responded did not attend formal education; 249 (61.3%), and more than half of the study participants (229, or 56.4%) were housewives by occupation, 244 (60.1%) of the respondents had either a TV and/or radio in their house (Table 1).

**Table 1:** Socio-demographic characteristics of mothers who give birth in Ayssaeta district, Afar, Northeast Ethiopia in 2020 (N=406)

Variables	Category	Frequency	percent
Mother's age	15–24 year	73	18.0
	25–34 year	233	57.4
	≥ 35 years	100	24.6
Residence	Rural	209	51.5
	Urban	197	48.5
Marital status	Married	385	94.8
	Others*	21	5.2
Religion	Muslim	376	92.6
	Orthodox	20	4.9
	Protestant	10	2.5
Mother's education	No education	249	61.3
	Primary	102	25.1
	Secondary and higher	55	13.6
Husband education (n=385)	No education	163	42.3
	Primary	65	16.9
	Secondary and higher	157	40.8
Mother's occupation	Housewife	229	56.4
	Pastoralist	98	24.0
	Argo-pastoralist	25	6.2
	Merchant	25	6.2
	Civil servant	17	4.2
	Others**	12	3.0
Partner's occupation (n=385)	Pastoralist	125	32.5
	Civil servant	97	25.2
	Merchant	77	20.0
	Argo-pastoralist	58	15.1
	Others***	28	7.3
Family size	3–5	194	47.8
	6–8	162	39.9
	≥9	50	12.3
Had radio and/or TV	No	244	60.1
	Yes	162	39.9
Decides on own healthcare	Mother and husband	154	37.9
	Husband only	110	27.1
	Mother only	95	23.4
	Others****	47	11.6

Others* = Single, widowed and **Divorced**; Others** = Daily laborer, Housemaid, Student and **Private implored**; Others*** = Daily laborer, Charcoal Work, and Unemployed; Others**** = Traditional Birth Attendants and Elders

**Obstetric characteristics**: Of 406 the study participants, 53.4% of mothers got married before the age of 18 years. The majority of the participants (47.8%) had 3–5 family members, and 39.9% of the respondents had 6–8 family sizes. Of all the respondents, 54.2% of women attended antenatal care (ANC) during their last pregnancy. Of all mothers, 53% had more than four live children. Less than half (46.6%) of the respondents gave birth to their last child at a health facility, and 45.1% of women were attendants at PNC in their last pregnancy 35.5% of the mothers faced postpartum complications (Table 2).

**Table 2:** Obstetrics characteristics of mothers who give birth in Ayssaeta district, Afar, Northeast Ethiopia 2020 (N=406)

Variables	Category	Frequency	Precent
Age at first marriage	< 18 years	217	53.4
	≥18 year	189	46.6
Gravidity	1–3	186	45.8
	4–6	156	38.4
	≥ 7	64	15.8
Number of living children	1–3	191	47.0
	≥ 4	215	53.0
Index pregnancy planned	No	226	55.7
	Yes	180	44.3
ANC during last pregnancy	No	186	45.8
	Yes	220	54.2
Number of ANC visits (n = 220)	1–3	194	88.2
	≥ 4	26	11.8
Gestational age at 1^st^ ANC	≤ 4 months	64	29.1
	≥ 5 months	156	70.9
Advices on PNC during ANC Visits	No	33	15.0
	Yes	187	85.0
ANC during last pregnancy	No	262	64.5
	Yes	144	35.5
Place of delivery (last child)	Home	217	53.4
	Health facility	189	46.6
Nature of delivery baby	Spontaneous	347	85.5
	Assisted	45	11.1
	Caesarean	14	3.4
Had Postpartum complications	No	262	64.5
	Yes	144	35.5
Ever attended PNC before	No	209	51.5
	Yes	197	48.5
Attended PNC visit last Pregnancy	No	223	54.9
	Yes	183	45.1
Number of PNC visit	1–3	158	86.3
	≥ 4	25	13.7
Advised during PNC follow-up	No	22	12.0
	Yes	161	88.0

**Factors associated with postnatal care utilization**: In multivariable logistic regression analysis, after controlling for potential confounders, residence, place of delivery, having labor and postpartum complications, and being advised about PNC during antenatal care visit were found to have a statistically significant association with the outcome variable. For instance, mothers who resided in urban areas were 3.69 times more likely to use postnatal-care services compared with those who resided in rural areas [AOR =3.69, 95% CI: 2.35–6.29]. Those mothers who had a health facility delivery and supported pregnancy for their last birth were 5.91 times more likely to utilize postnatal-care services than those who had a home delivery [AOR =5.91, 95% CI: 4.68–9.34]. Mothers who had complications during PNC care services were 3.13 times more likely to utilize postnatal care compared with those who had no complications during PNC care services [AOR =3.13, 95% CI: 2.26–5.17]. Mothers who had gotten advice about PNC during ANC visits were 3.74 times more likely to utilize postnatal-care services compared with those who had received no advice about PNC during ANC visits [AOR =3.74, 95% CI: 1.17–11.95] (Table 3).

**Table 3:** Bivariable and multivariable logistic regression analysis of the factors affecting postnatal care utilization among mothers in Ayssaeta district, Northeast Ethiopia, 2020

Variables	PNC Utilization		COR (95% CI)	AOR (95% CI)

	Yes	No		
**Residence**				
Rural	82(44.8)	127(57.0)	1	1
Urban	101(55.2)	96(43.0)	1.63(1.10,2.42)	**3.69(2.35, 6.29)** *
**Radio and/or TV**				
No	80(43.7)	164(73.5)	1	1
Yes	103(56.3)	59(26.5)	3.58(2.36, 5.43)	2.24(0.81,6.19)
**Number of ANC visits**				
1–3	117(86.7)	77(90.6)	1	1
≥4	18(13.3)	8(9.4)	1.48(0.61, 3.57)	1.07(0.27,4.17)
**Gestation age at 1^st^ ANC**				
< 4 moths	48(35.6)	16(18.8)	1	1
≥4 months	87(64.4)	69(81.2)	0.42(0.22, 0.80)	0.65(0.24, 1.77)
**Advised about PNC during** **ANC visits**				
No	14(8.6)	19(19.1)	1	1
Yes	149(91.4)	38(80.9)	5.27(3.38, 8.22)	**3.74(1.17,11.95)** *
**Index pregnancy planned**				
No	91(49.7)	135(60.5)	1	1
Yes	92(50.3)	88(49.5)	1.55(1.05,2.30)	0.92(0.37, 2.32)
**Place of delivery**				
Home	76(39.4)	141(66.2)	1	1
Health facility	117(60.6)	72(34.8)	3.06(2.61, 6.03)	**5.91(4.68, 9.34)** *
**Had pregnancy complication**				
No	98(53.6)	164(73.5)	1	1
Yes	85(46.4)	59(26.5)	2.41(1.59,3.65)	1.44(0.58, 3.56)
**Had labour and postpartum** **complication**				
No	72(39.3)	184(82.5)	1	1
Yes	111(60.7)	39(17.5)	7.27(4.61, 11.67)	**3.13(2.26, 5.17)** *

COR = Crude Odds Ratio, AOR = Adjusted odds ratio, ANC=Antenatal care

* Statistically significant variables at *P*-value < 0.05

## Discussion

This study revealed that the proportion of mothers who had attended at least one postnatal care visit was 45.1% (95% CI: 40.0%–50.0%). This finding was slightly comparable to reports from studies conducted in Halaba, Southern Ethiopia 47.9% (21), and Malawi 48.4% (22). On the contrary, this finding is lower than those of studies conducted in some other African countries: Ethiopia(20, 23, 24), Tanzania 70.8% (25). The possible reason for this difference might be due to a low awareness level of postnatal care availability among women in study area(12). It might also be due to widespread cultural and spiritual taboos and the traditional beliefs and lifestyles of the study community. Furthermore, women may not seek health care due to social and traditional perceptions as well as a lack of women's power to make independent decisions to seek health care. (13, 26).

This study's results were higher compared with studies done in Ethiopia, which found a range of 6 percent to 37 percent (27–31) and a study conducted in rural districts in four Sub-Saharan African countries :Burkina Faso 25%, Kenya 33%, Malawi, 41%, and Mozambique 40% (32). This may be attributed to the time difference in some studies since there could be an improvement in accessing and utilizing health care services over time. Other possible explanations for these discrepancies include a year-to-year rise in government efforts to promote mother and newborn health as well as differences in location and social context between the studies (12, 16).

On the other hand, these study findings were higher than 2019 EDHS reports for the Afar region and country (11). The other possible reason for the study of EDHS was that it included women who lived in urban and rural areas of the country, but this study included only three rural and one urban participant. As a result, women who live in these study areas may have a better chance of receiving information on PNC opportunities and maternal health care services, due to the sample size and the time difference between studies is another possibile justification.

This study also revealed that the odds of PNC service utilization among women who gave birth at a health facility were 5.91-fold higher compared to women who delivered their last birth at home. This finding is consistent with the findings of the studies conducted in different parts of Ethiopia (20, 24, 27, 28, 30, 33), and various areas in Sub-Saharan African countries (34). The possible explanation is that women who give birth in health facilities have a better opportunity to get health education regarding postnatal care services and have access to benefits and availability of PNC services during their stay in health facilities. This study's findings showed that residence had an effect on postnatal care service utilization. Mothers in urban areas were 3.69 times more likely to seek postnatal care than those in rural areas. This study's finding is supported by previous studies that have shown a positive association between urban residency and postnatal care service utilization.(6, 34).

The discrepancy may be explained by the physical proximity to health facilities, insufficient transportation, remote nature, and hot weather conditions of reaching a health facility in rural areas (13), as well as cultural and traditional practices and customs more prevalent in rural areas than in urban areas, which are barriers to using delivery services. (26).

Additionally, compared with women living in rural areas, urban areas generally have better access to PNC services as well as other advantages of urban life, such as greater exposure to health promotion programs(35). Women in rural areas are less likely to use PNC, especially when giving birth in a health center, than women in cities. This makes their chances of getting sick or dying higher. Mothers who had delivery complications after giving birth were 3.13 times more likely to seek postnatal care services than mothers who did not face any complications. The findings resembled those of previous research in Ethiopia's Wonago district and Debre Markos town, as well as Nepal (30, 36, 37). It's possible that being exposed to problems increases the fear of developing more; women who have seen some warning signals have a more negative impression of vulnerability and the severity of outcome risks, leading to increased postnatal care utilization and, due to awareness of maternal complications, maybe a significant motivator for women and their families to seek care as early as possible. Another possible reason could be healthcare providers' improvement in preemptively consulting mothers who experienced complications during delivery and advising them on the necessity of PNC use and following delivery to prevent complications and follow-up mothers.

The odds of receiving post-natal care were higher among women who received advice during antenatal care visits compared with women who did not received advice during antenatal care visits. This finding complemented previous studies (38, 39). This could be due to the fact that attending antenatal care services gives pregnant women the chance to learn about possible preparations for childbirth and the benefits of giving birth in a health facility. They also usually learn about the importance of using PNC service along with ANC examination maternal health care service linkage. However, the results of other studies (40) showed that antenatal consultations were not associated with the uptake of PNC, which might be due to mothers not being told about the necessity of PNC; its availability; recommended timing; and targeted frequency of postnatal visits, during their antenatal appointment and before being discharged.

The possibility of recall bias is due to the fact that the women were asked about events within the last year prior to the study. As the survey asked for information retrospectively, this may have yielded some recall bias. To minimize recall bias, we restricted gathered information to the last one year per study birth. This study's design is quantitative and doesn't address the cultural issues of the respondents. As a strength, it is one of the few postnatal care services studies conducted in pastoralist communities. This study may also provide policymakers with insight into the factors that influence community postnatal care service utilization, as well as plans for service enhancement..

In conclusion, this study found that postnatalcare service utilization is low in the study area, but it is higher than in the previous regional and national EDH reports. This study revealed residency, place of delivery, labor and postpartum complications, and prenatal care counseling during ANC visits as vital determinants of PNC service consumption among study participants.Enhance postnatal care use by strengthening postnatal care strategies, increasing and sustaining utilization of healthcare delivery and special attention to rural mothers. Further qualitative research needs to be done to find out what kinds of cultural beliefs might stop women from getting PNC services during the postpartum period.
